# Protein Interaction Z Score Assessment (PIZSA): an empirical scoring scheme for evaluation of protein–protein interactions

**DOI:** 10.1093/nar/gkz368

**Published:** 2019-05-22

**Authors:** Ankit A Roy, Abhilesh S Dhawanjewar, Parichit Sharma, Gulzar Singh, M S Madhusudhan

**Affiliations:** 1 Indian Institute of Science Education and Research, Pune, Dr Homi Bhabha Road, Pashan, Pune 411008, India; 2 presently at School of Biological Sciences, University of Nebraska, Lincoln, NE 68588, USA; 3 presently at School of Informatics, Computing & Engineering, Department of Computer Science, Indiana University, Bloomington, IN 47408, USA

## Abstract

Our web server, PIZSA (http://cospi.iiserpune.ac.in/pizsa), assesses the likelihood of protein–protein interactions by assigning a Z Score computed from interface residue contacts. Our score takes into account the optimal number of atoms that mediate the interaction between pairs of residues and whether these contacts emanate from the main chain or side chain. We tested the score on 174 native interactions for which 100 decoys each were constructed using ZDOCK. The native structure scored better than any of the decoys in 146 cases and was able to rank within the 95th percentile in 162 cases. This easily outperforms a competing method, CIPS. We also benchmarked our scoring scheme on 15 targets from the CAPRI dataset and found that our method had results comparable to that of CIPS. Further, our method is able to analyse higher order protein complexes without the need to explicitly identify chains as receptors or ligands. The PIZSA server is easy to use and could be used to score any input three-dimensional structure and provide a residue pair-wise break up of the results. Attractively, our server offers a platform for users to upload their own potentials and could serve as an ideal testing ground for this class of scoring schemes.

## INTRODUCTION

Protein-protein interactions (PPIs) are key to almost all cellular functions. In order to better appreciate and/or get insights into these cellular processes, it is crucial that we discover the entire network of underlying interactions. Determining protein interacting partners experimentally is both, time-consuming and expensive. Also, given the magnitude of the problem, where estimates say there could be upto 130,000 PPIs among the Human proteome alone (1–3), it is imperative that we develop computational techniques to predict such interactions (4–6).

Computational predictions usually consider geometric and chemical complementarity of the interactors to suggest viable PPIs. Typically, the problem involves sampling various conformations of the interactors (with respect to one another) and then scoring each of these proposed associations. In this manuscript, we present a platform for scoring putative PPIs demonstrated with our newly developed statistical potential that builds on an older formulation (7). Our scoring scheme, dubbed PIZSA for Protein Interaction Z-Score Assessment, utilizes the fact that large observed/expected ratios are indicative of favorable energetics. Particularly, PIZSA makes use of pairwise associations of amino acids that are in close physical proximity across the PPI interface.

Our scoring scheme involves the computation of three matrices of amino acid pair preferences for interactions mediated by main chain–main chain, main chain–side chain and side chain–side chain atomic contacts. We explored and optimized values for the most effective contact distance based on a benchmark (8). We have also compared ourselves with another method and demonstrated that we perform at the same level if not better at identifying native structures among a set of decoys (8–10).

In the analysis of our scoring scheme presented here, we have compared its performance on two datasets of native PPIs and decoys (11–14). We have compared our performance to that of CIPS (10), which in turn was already shown to outperform three other methods (15–17). We further demonstrate PIZSA’s usability in assessing protein–protein interfaces through two case studies. Particularly, one of the crucial aspects of the way we have constructed the scoring scheme is to enable the same evaluation to be carried out for all oligomeric states and not restrict ourselves to dimeric interactions.

## MATERIALS AND METHODS

### The PIZSA scoring scheme to assess protein complexes

Interacting residue pairs on the interface of protein–protein complexes are identified based on a distance threshold. Residues belonging to different chains of a multi-chain protein complex that are within a distance threshold of each other are identified as interacting residue pairs. Users can choose 4, 6 or 8 Å as the distance threshold that is otherwise set to 4 Å by default. Interacting residue pairs are assigned three different scores depending on the types of atoms involved in the interaction (main chain - main chain, main chain - side chain and side chain - side chain). Every residue pair score is further normalized by their atomic propensities and a clash penalty. Atomic propensities are a measure of how frequently a specific number of atoms are involved in a residue pair interaction and clash penalties are used to penalize interactions with steric clashes. The final score assigned to a protein–protein complex is the sum of all the scores assigned to its interacting residue pairs. Every protein complex is also assigned a *Z* score that predicts the likelihood of a protein complex to form a stable association by comparing its score to a background distribution of scores obtained from non-native interactions. Protein complexes are predicted to form stable associations if they acquire *Z* scores above certain thresholds. *Z* score thresholds have been optimized at different distance cut-offs, the details of which along with the details of our scoring functions are reported elsewhere (8).

### Identification of native complexes and stable associations

The ability of our method to identify the native complex amongst a set of decoys was tested on the ZDOCK Docking Benchmark 4.0 (11,12) and CAPRI (13,14) decoy sets. We used ZDOCK 3.0.2 with 6-degree sampling to generate 100 decoys for each of the 174 targets. We also used decoys from 9 separate CAPRI rounds (R8, R9, R10, R27, R28, R29, R31, R32 and R34) that include 15 targets (T22, T23, T24, T25, T26, T58, T61, T67, T95, T98, T99, T100, T101, T104 and T105) and ∼5000 decoys. All decoys and native complexes were evaluated using the PIZSA and CIPS potentials. Further, complexes were rank-ordered according to their respective scores and ranks obtained by the native complexes with PIZSA and CIPS potentials were compared. We also tested the ability of PIZSA to classify native complexes as stable associations on the ZDOCK decoy set. Classification performance was tested by evaluating the accuracy, balanced accuracy and the Matthews correlation coefficient (MCC).

## RESULTS

### Performance on ZDOCK Docking Benchmark 4.0

**Figure 1.**
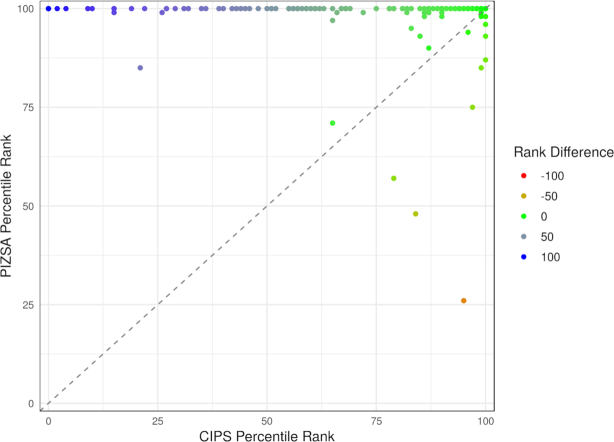
The percentile ranks of native protein–protein complexes in the ZDOCK Docking Benchmark 4.0 as calculated using PIZSA (y-axis) and CIPS (x-axis) respectively. Points on the diagonal are instances where both PIZSA and CIPS assign equal ranks (green). Points above the diagonal are cases where PIZSA assigned a better rank to the native complex (progressively bluer shades). Points below the diagonal are cases where CIPS assigned a better rank to the native complex (progressively redder shades).

We tested our method’s ability to score native protein complexes optimally on the ZDOCK Docking Benchmark 4.0 and compared our performance with that of CIPS. Of the 174 native protein complexes, we were able to correctly identify the native structure as the best scoring in 146 cases (84%) whereas CIPS identified the native to be the best in only 26 cases (15%). Furthermore, our method ranked native interactions among the top three interactions in 160 cases (92%), among the top 5 interactions in 162 cases (93%) and among the top 10 interactions in 166 cases (95%) whereas CIPS ranked the native complexes among the top 3, 5 and 10 interactions in 50 (29%), 59 (34%) and 79 (45%) cases respectively (Table 1). PIZSA ranks the native interaction better than CIPS in 140 cases (81%), equal to that of CIPS in 23 cases (13%) and worse than that of CIPS in 11 cases (6%) (Figure 1, Supplementary S1).

**Table 1.** Comparison of the number of native target complexes ranked as top scoring interactions

Rank (in top)	PIZSA	CIPS
1	146	26
3	160	50
5	162	59
10	166	79

We also tested our method’s ability to classify native protein–protein interactions as stable associations using a *Z* Score assessment. Since the dataset has a larger representation of the negatives as compared to the positives (100 decoys for a single native complex), we report our performance in terms of rates instead of absolute numbers. We were able to correctly predict 158 of 174 native structures as stable associations at a distance threshold of 4 Å and a *Z* Score threshold of 1.5 with a true positive rate of 0.91, true negative rate of 0.89, false positive rate of 0.11 and false negative rate of 0.09. PIZSA achieved a classification accuracy of 0.89, balanced accuracy of 0.90 and an MCC of 0.80 (calculated using rates, Supplementary S2).

### Performance on CAPRI targets

We tested our method’s ability to identify native structures among a set of decoys in different CAPRI rounds. 15 CAPRI targets were evaluated of which 12 were dimeric complexes and 3 were higher order associations (>2 chains). PIZSA was able to identify the native complex in the top 10% of the decoys in six cases and in the top 20% of the decoys in 10 cases. We compared the ranks of native complexes assigned by PIZSA with those assigned by CIPS (Table 2). CIPS was able to identify the native complexes in the top 10% of decoys for five cases and in top 20% of the decoys in eight cases of the 12 evaluated targets. PIZSA assigned a rank better than or equal to that of CIPS in five cases whereas CIPS assigned a rank better than PIZSA in 7 cases. However, three multimeric targets (T95, T99 and T100) were not evaluated using CIPS as it requires interactions to be dimeric, whereas, PIZSA can evaluate quaternary associations of multiple structures as a whole and does not require an explicit demarcation of receptor and ligand. Although CIPS assigns better ranks to native complexes in more cases than PIZSA, the overall difference in ranking among the two methods is statistically insignificant (*P*-value of 0.5 on a Wilcoxon signed-rank test).

**Table 2.** Percentile ranks of native complexes from CAPRI targets as evaluated using PIZSA and CIPS

CAPRI Target	PDB ID	Number of decoys	PIZSA rank of native	CIPS rank of native
T22	2J59	90	88	69
T23	2J59	98	90	74
T24	2HQS	355	97	98
T25	2ONI	396	95	98
T26	4AK2	426	77	84
T58	4G9S	230	71	84
T61	3ZIO	270	95	95
T67	4N7H	351	42	55
T95	4R8P	240	80	*
T98	4UEM	418	87	100
T99	4UEL	399	97	*
T100	4UF6	380	71	*
T101	4UF5	398	98	97
T104	4UHP	496	56	61
T105	4QKO	506	82	81

Unevaluated targets are indicated by * in the rank field.

## WEB SERVER DESCRIPTION

The PIZSA web server provides an easy-to-use graphical interface for the assessment of the stability of quaternary protein assemblies. The web server is freely accessible without login requirements at http://cospi.iiserpune.ac.in/pizsa/. Users can either specify the four-letter PDB code of dimeric or multimeric protein complexes or upload a complex as a single file in PDB format. They have the option of choosing between three distance thresholds (4, 6 and 8 Å) to define amino acid interactions and also of using their own custom scoring matrices for different modes of interactions. The default settings for PIZSA utilize the pairwise amino acid preferences defined for all atom types at a distance threshold of 4 Å. The server also has a provision for carrying out mutational analysis. In the current version of the server, this utility has not yet been optimized and is currently being tested and refined and as such the results produced by this module is outside the consideration of this study. Copious help pages guide users through the website. Finally, users of the web server also have the option of providing an email address to receive notifications especially for computationally intensive analyses.

## WEB SERVER OUTPUT

**Figure 2.**
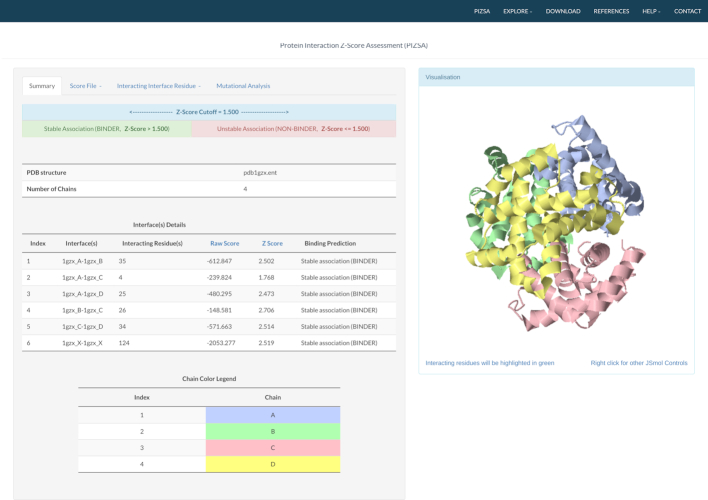
PIZSA web server output for the evaluation of the tetrameric Haemoglobin complex (PDB code: 1GZX). Summary tab displays the number of interacting residues, Raw Score and *Z* Score of interaction along with a binding prediction of stable association for all possible interfaces. The Haemoglobin complex is rendered as a ribbon diagram with color-coded chains.

The output from PIZSA is presented in four tabs; ‘Summary’, ‘Score File’, ‘Interacting Interface Residues’ and ‘Mutational Analysis’. The first tab summarises the results of evaluating the query protein complex. It displays the Raw Score and the *Z* Score obtained by the protein complex and also whether or not the complex was predicted as a stable association (Figure 2). The ‘Score File’ tab provides a table listing the different parameters used during the analysis and the resulting scores (Raw Score, Normalized Raw Score and *Z* Score) for the input protein complex. Individual scores for every interacting residue pair are reported in the ‘Interacting Interface Residues’ tab. All interface residues are listed by the residue number, residue name, the polypeptide chain that they belong to, their interacting partner and their interaction score. Users have the option to filter this list by searching for specific residue names or residue numbers. Hovering the cursor over this list highlights the interacting residue pairs on the structure that is displayed by default as a ribbon diagram on the right side of the window and is color-coded to distinguish different chains. Since the protein complex is rendered using JSmol (18), users are free to interact with the structure and tweak the visuals as per their liking. The results of an *in silico* mutational analysis are provided on the ‘Mutational Analysis’ tab. Data from the Score File, Interacting Interface Residues and Mutational Analysis can be downloaded as separate files. A stand-alone version of PIZSA can be downloaded from the download link provided on the web server.

## CASE STUDIES

### The Ras:SOS:Ras ternary complex

**Figure 3.**
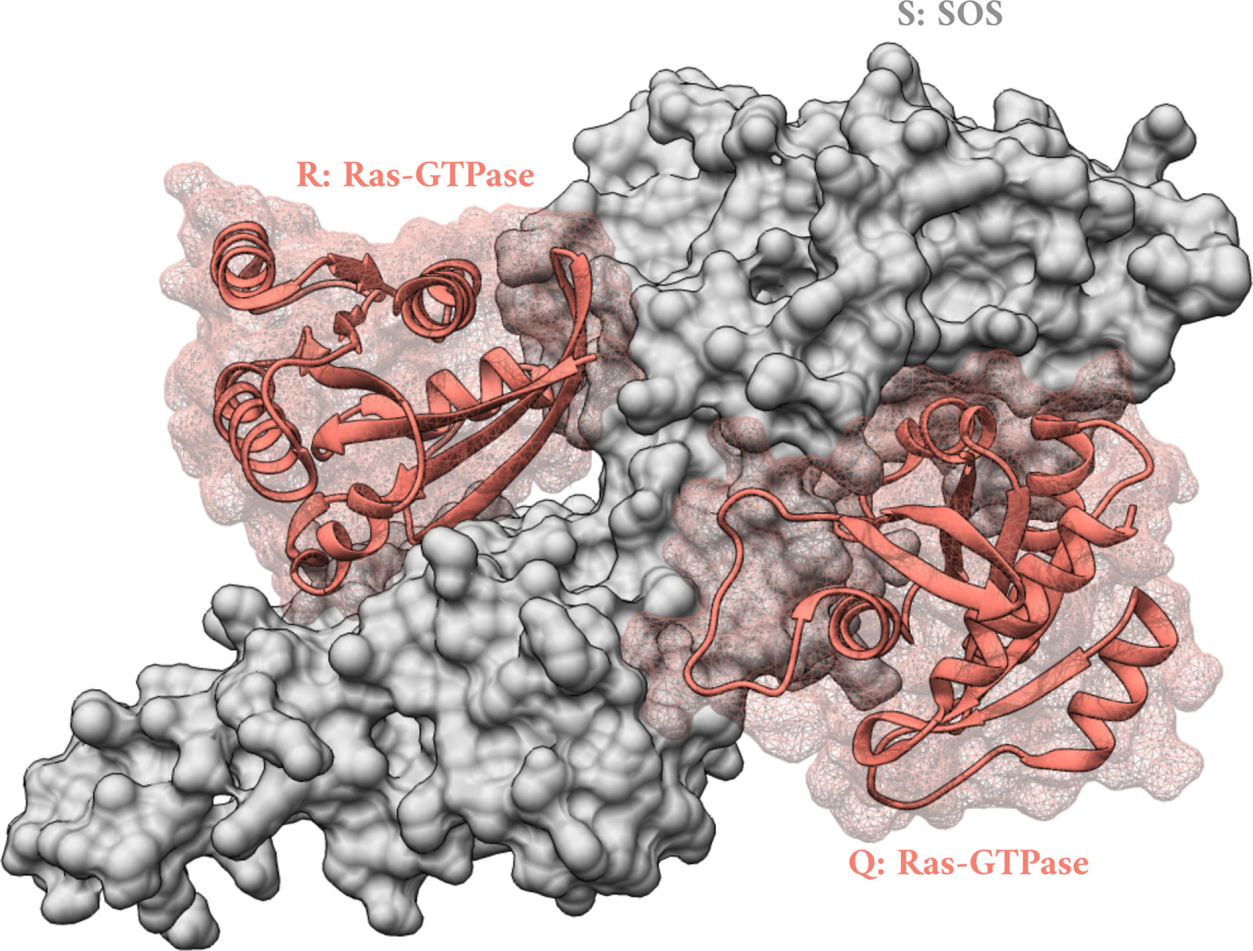
The structure of Ras:SOS:Ras ternary complex (PDB code: 1NVV). The structure of SOS (S) has been represented with a solvent-excluded surface in grey and Ras-GTPases (Q and R) have been represented as ribbons (salmon) with meshed surfaces. Representations were rendered using UCSF Chimera (21).

Many a times, a set of proteins interact with each other in more than one biologically meaningful way (19). An example of this is the interaction between the Ras GTPase and the nucleotide exchange factor Son Of Sevenless (SOS). Ras catalyzes the dephosphorylation of guanosine triphosphate to guanosine diphosphate (GTP to GDP). SOS is required for release of GDP from the Ras-GDP complex. It has been shown previously that Ras-GTP binds on a site orthogonal to that of the SOS:Ras-GDP interface and accelerates the release of GDP. Therefore, SOS has two spatially-separated Ras binding sites and can form a ternary complex binding both Ras-GDP and Ras-GTP (PDB code: 1NVV, Figure 3) (20). We used PIZSA to evaluate the two orthogonal SOS:Ras (interfaces QS and RS) interactions and the ternary complex Ras:SOS:Ras. PIZSA classifies both the dimeric SOS:Ras interactions as well as the ternary complex as binders (Table 3). We constructed alternate docking conformations for the SOS:Ras dimers using the ZDOCK web server (22). The top 10 predicted poses for each SOS:Ras dimer were all predicted as non-binders when evaluated using PIZSA (Supplementary S3).

**Table 3.** Evaluation of Ras:SOS dimeric (QS and RS) and Ras:SOS:Ras trimeric complexes with PIZSA

Protein complex	Number of interacting pairs	Normalized raw score	Z Score	Binding prediction
Ras:SOS:Ras				
Trimer	139	−22.94	2.40	Binder
Ras:SOS (RS)				
dimer	70	−24.99	2.37	Binder
SOS:Ras (QS)				
dimer	69	−20.85	2.44	Binder

### The quaternary structure of Haemoglobin

The Human haemoglobin has a tetrameric structure consisting of two α-globin subunits with 141 amino acids each and two β-globin subunits with 146 amino acids each. The quaternary structure of haemoglobin can be considered as a dimer of two αβ dimers (α_1_β_1_ and α_2_β_2_) that are rotated by 180^○^ with respect to each other and acquire two-fold symmetry around a central water-filled cavity. The haemoglobin molecule can exist in two states: oxygenated (or R-state) and deoxygenated (or T-state). Upon the binding of oxygen, the haemoglobin molecule transitions from the T-state to the R-state undergoing a conformational change in which the α_2_β_2_ dimer rotates ∼15^○^ relative to the α_1_β_1_ (23). This T to R-structure transition also leads to the disruption of old and generation of new interactions among the amino acid residues in the α_1_β_2_ and α_2_β_1_ interfaces and corresponds to a free energy difference of approximately 6 kcal/mole between the deoxy-haemoglobin tetramer and the oxy-haemoglobin tetramer (−14 and −8 kcal/mol respectively) arising from more extensive interactions in the deoxy-haemoglobin (24).

We used PIZSA to evaluate the stability of the two tetrameric conformations of the haemoglobin molecule. We correctly classified both tetramers as binders. The deoxygenated state is thermodynamically more stable than the oxygenated state due to the presence of more extensive amino acid interactions in the deoxygenated haemoglobin. *Z* Scores assigned to these states are in agreement with their observed thermodynamic stabilities (Table 4). This is expected as higher *Z* Scores are associated with more stable assemblies as per our scoring scheme (8).

**Table 4.** PIZSA scores for the hemoglobin complexes in the oxygenated (PDB code: 1GZX) and the deoxygenated (PDB code: 2HBB) states

Parameters	Oxygenated Hb	Deoxygenated Hb
	(R-state)	(T-state)
Number of interacting pairs	124	123
Raw score	−2053.27	−1243.68
Normalized raw score	−16.55	−10.11
*Z*-Score	2.51	2.60

## DISCUSSION AND CONCLUSION

We have developed a knowledge-based statistical potential that evaluates protein complexes based on the distribution and type of pairwise residue contacts at the interface. Our method explicitly accounts for the separate contributions of different modes of interaction between contacting residue pairs (main chain–main chain, main chain–side chain and side chain–side chain). Such a distinction is necessary as the same interacting residue pairs have varying preferences for different modes of interactions (8). Interacting residue pairs are defined on the basis of distance thresholds that can be toggled by the user to account for short range direct interactions at 4 Å to progressively longer range interactions at 6 and 8 Å such as water mediated interactions (25).

Amino acid pairs on the interface are scored based on their pairing preferences and their tendencies to interact with a certain number of atoms (8). These preferences have been quantified in the form of 6 scoring matrices for every distance threshold and can be customized at the user’s discretion. Our web server allows modularity in uploading one or more custom scoring matrices alongside the default ones, essentially allowing users to make use of our platform to test various other scoring matrices. PIZSA not only provides a cumulative score for all the pairwise interactions on an interface but also classifies protein complexes as stable or unstable assemblies. We have benchmarked our classification performance on the ZDOCK decoy set with an accuracy of 0.89, balanced accuracy of 0.90 and an MCC of 0.80. We have also previously benchmarked our classification with similar levels of performance on the Dockground Docking Decoy Set (8).

We demonstrated the ability of PIZSA to identify native complexes and compared it with that of CIPS on two different datasets, the ZDOCK Docking Benchmark 4.0 and the CAPRI decoy set. Our method identified 146 out of 174 native complexes as the best interaction in the ZDOCK data set and outperformed CIPS that was able to identify only 26 native complexes as the best interactions. On the CAPRI dataset, both methods had comparable results, however CIPS can only score multimeric assemblies if they are bifurcated into chains for receptors and chains for ligands. The CAPRI dataset consists of many more decoys that are close to the native structure, or in the immediate proximity of the true binding site, than the ZDOCK decoys. This follows from the method ZDOCK uses to create the decoys - by design 5/6 of the complexes have non-native contact while the other 1/6 explore different conformations in the true binding site. We believe that in comparison to CIPS, PIZSA can better discern between completely non-native structures and native like structures.

PIZSA successfully identified the native complexes of a dodecamer (T95: 4R8P), and two trimers (T99: 4UEL and T100: 4UF6) with percentile ranks of 80, 97 and 71 respectively. Our method can potentially be used to assess models of higher order multimeric protein complexes such as those constructed by fitting monomeric subunits onto electron density maps (26–28).

We have demonstrated the utility of PIZSA using two case studies. In the first of the two, we show that PIZSA is able to identify alternative binding modes of Ras with SOS along with the Ras:SOS:Ras ternary complex. In the second case study we demonstrate that our scores concur with experimental observations in suggesting that the deoxygenated tetrameric complex of Haemoglobin is more stable than its oxygenated counterpart.

## Supplementary Material

gkz368_Supplemental_Files
