# Supplementary material for: Protein Interaction Z Score Assessment (PIZSA): an empirical scoring scheme for evaluation of protein–protein interactions
Source: Nucleic Acids Res. 2019 May 22;47(W1):W331–7. doi: 10.1093/nar/gkz368 (PMC6602501; doi:10.1093/nar/gkz368)
Supplement: gkz368_Supplemental_Files [file gkz368_supplemental_files.pdf]

Supplementary Data:

Protein Interaction Z Score Assessment (PIZSA): an empirical scoring scheme for evaluation of protein-protein interactions

Ankit A. Roy, Abhilesh S. Dhawanjewar, Parichit Sharma, Gulzar Singh, and M.S. Madhusudhan

Supplementary S1. Native complex percentile ranks of ZDOCK Docking Benchmark 4.0 targets evaluated using PIZSA and CIPS respectively.

PDB code	PIZSA Rank	CIPS Rank	Rank Difference
1A2K	100	100	0
1ACB	97	65	32
1AHW	100	57	43
1AK4	100	61	39
1AKJ	100	43	57
1ATN	98	86	12
1AVX	96	100	-4
1AY7	100	9	91
1AZS	100	100	0
1B6C	100	92	8
1BGX	100	0	100
1BJ1	100	78	22
1BKD	100	65	35
1BUH	100	96	4
1BVK	100	68	32
1BVN	99	66	33
1CGI	100	93	7
1CLV	100	57	43
1D6R	100	81	19
1DE4	100	100	0
1DFJ	100	85	15
1DQJ	100	88	12
1E4K	85	99	-14
1E6E	100	40	60
1E6J	100	79	21
1E96	100	19	81
1EAW	100	10	90

Continued on next page

Table continued from previous page

PDB code	PIZSA Rank	CIPS Rank	Rank Difference
1EER	100	96	4
1EFN	100	90	10
1EWY	100	94	6
1EZU	100	56	44
1F34	100	93	7
1F51	99	15	84
1F6M	100	100	0
1FAK	100	91	9
1FC2	94	96	-2
1FCC	93	85	8
1FFW	100	67	33
1FLE	99	86	13
1FQ1	100	89	11
1FQJ	100	0	100
1FSK	100	69	31
1GCQ	99	82	17
1GHQ	100	4	96
1GL1	100	56	44
1GLA	100	59	41
1GP2	100	99	1
1GPW	100	63	37
1GRN	100	100	0
1GXD	100	95	5
1H1V	26	95	-69
1H9D	100	39	61
1HCF	100	57	43
1HE1	100	61	39
1HE8	75	97	-22
1HIA	100	44	56
1I2M	100	52	48
1I4D	100	95	5
1I9R	100	0	100
1IB1	100	27	73
1IBR	100	96	4
1IJK	100	90	10
1IQD	100	96	4
1IRA	93	100	-7
1J2J	100	99	1
1JIW	100	89	11
1JK9	100	61	39
1JMO	98	99	-1
1JPS	100	15	85
1JTG	100	68	32
1JWH	99	99	0
1JZD	100	100	0

Continued on next page

Table continued from previous page

PDB code	PIZSA Rank	CIPS Rank	Rank Difference
1K4C	48	84	-36
1K5D	100	97	3
1K74	100	78	22
1KAC	100	97	3
1KKL	100	59	41
1KLU	100	100	0
1KTZ	100	94	6
1KXP	100	100	0
1KXQ	100	75	25
1LFD	100	100	0
1M10	99	87	12
1MAH	100	67	33
1ML0	98	90	8
1MLC	100	90	10
1MQ8	100	90	10
1N2C	100	90	10
1N8O	100	45	55
1NCA	100	35	65
1NSN	100	94	6
1NW9	100	81	19
1OC0	100	100	0
1OFU	100	100	0
1OPH	100	55	45
1OYV	100	100	0
1PPE	100	58	42
1PVH	100	42	58
1PXV	100	78	22
1QA9	57	79	-22
1QFW	87	100	-13
1R0R	100	87	13
1R6Q	100	99	1
1R8S	100	99	1
1RLB	100	94	6
1RV6	100	100	0
1S1Q	100	2	98
1SBB	100	0	100
1SYX	99	26	73
1T6B	100	55	45
1TMQ	100	32	68
1UDI	100	86	14
1US7	100	62	38
1VFB	100	51	49
1WDW	100	68	32
1WEJ	100	97	3
1WQ1	100	99	1

Continued on next page

Table continued from previous page

PDB code	PIZSA Rank	CIPS Rank	Rank Difference
1XD3	100	90	10
1XQS	99	72	27
1XU1	100	94	6
1Y64	85	21	64
1YVB	100	98	2
1Z0K	100	83	17
1Z5Y	100	97	3
1ZHH	100	94	6
1ZHI	100	97	3
1ZLI	90	87	3
1ZM4	100	98	2
2A5T	100	0	100
2A9K	100	55	45
2ABZ	100	95	5
2AJF	100	98	2
2AYO	100	97	3
2B42	100	0	100
2B4J	100	50	50
2BTF	100	78	22
2C0L	100	22	78
2CFH	100	98	2
2FD6	98	100	-2
2FJU	100	100	0
2G77	100	48	52
2H7V	95	83	12
2HLE	100	31	69
2HMI	100	100	0
2HQS	100	60	40
2HRK	100	79	21
2I25	100	100	0
2I9B	100	100	0
2IDO	100	100	0
2J0T	100	46	54
2J7P	100	27	73
2JEL	100	97	3
2MTA	100	36	64
2NZ8	100	67	33
2O3B	100	100	0
2O8V	100	99	1
2OOB	100	0	100
2OOR	100	100	0
2OT3	100	78	22
2OUL	100	94	6
2OZA	99	90	9
2PCC	100	100	0

Continued on next page

Table continued from previous page

PDB code	PIZSA Rank	CIPS Rank	Rank Difference
2SIC	100	99	1
2SNI	100	97	3
2UUY	100	93	7
2VDB	100	99	1
2VIS	71	65	6
2Z0E	100	87	13
3BP8	100	82	18
3CPH	100	100	0
3D5S	100	87	13
3SGQ	100	29	71
4CPA	100	51	49
7CEI	100	15	85

Supplementary S2. Measures used for assessment of PIZSA classification performance. Classification performance was tested on the ZDOCK Docking Benchmark 4.0 with 174 native complexes (positives) and 17,400 decoy complexes (negatives). Native complexes classified as stable or unstable associations are identified as true positives (TP = 158, native complexes with Z Score $>$ 1.5) or false negatives (FN = 16, native complexes with Z Score $<=$ 1.5) respectively. Decoy complexes classified as stable or unstable associations are identified as false positives (FP = 1,963, decoy complexes with Z Score $>$ 1.5) or true negatives (TN = 15,437, decoy complexes with Z Score $<=$ 1.5) respectively. PIZSA classified the complexes with a True Positive Rate (1) of 0.91, False Negative Rate (2) of 0.09, True Negative Rate (3) of 0.89, False Positive Rate (4) of 0.11, Accuracy (5) of 0.89, Balanced Accuracy (6) of 0.90 and Matthews Correlation Coefficient, calculated using rates (7), of 0.80.

$$\mathrm{TPR} = \frac{\mathrm{TP}}{\mathrm{TP} + \mathrm{FN}} \tag{1}$$

$$\mathrm{FNR} = \frac{\mathrm{FN}}{\mathrm{TP} + \mathrm{FN}} \tag{2}$$

$$\mathrm{TNR} = \frac{\mathrm{TN}}{\mathrm{TN} + \mathrm{FP}} \tag{3}$$

$$\mathrm{FPR} = \frac{\mathrm{FP}}{\mathrm{TN} + \mathrm{FP}} \tag{4}$$

$$\mathrm{ACC} = \frac{\mathrm{TP} + \mathrm{TN}}{\mathrm{TP} + \mathrm{FP} + \mathrm{TN} + \mathrm{FN}} \tag{5}$$

$$\mathrm{BACC} = \frac{\mathrm{TPR} + \mathrm{TNR}}{2} \tag{6}$$

$$\mathrm{MCC} = \frac{\mathrm{TPR} \times \mathrm{TNR} - \mathrm{FPR} \times \mathrm{FNR}}{\sqrt{(\mathrm{TPR} + \mathrm{FPR})(\mathrm{TPR} + \mathrm{FNR})(\mathrm{TNR} + \mathrm{FPR})(\mathrm{TNR} + \mathrm{FNR})}} \tag{7}$$

		Interacting residue pairs	Normalized Raw Score	Z Score
**Native**		70	-24.99	2.37
**ZDOCK docking predictions**	1	74	-97.49	1.11
	2	57	-172.65	-0.18
	3	66	-105.994	0.97
	4	56	-144.06	0.31
	5	70	-166.95	-0.081
	6	43	-118.41	0.75
	7	45	-149.97	0.21
	8	59	-116.69	0.78
	9	47	-164.36	-0.03
	10	49	-155.54	0.11

Supplementary S3.A: PIZSA scores for various docking results of ZDOCK predictions for SOS:Ras (RS) interface.

		Interacting residue pairs	Normalized Raw Score	Z Score
**Native**		69	-20.85	2.44
**ZDOCK docking predictions**	1	48	-152.39	0.17
	2	40	-181.65	-0.33
	3	37	-125.7	0.63
	4	26	-132.89	0.5
	5	44	-204.5	-0.73
	6	49	-103.34	1.01
	7	30	-192.36	-0.52
	8	49	-132.03	0.52
	9	32	-125.18	0.64
	10	44	-98.83	1.09

Supplementary S3.B: PIZSA scores for various docking results of ZDOCK predictions for SOS:Ras (QS) interface.
